# Edible insect-based feed ingredients as modulators of gastrointestinal physiology in monogastric livestock and aquaculture species

**DOI:** 10.3389/fphys.2026.1945787

**Published:** 2026-10-23

**Authors:** Oscar Abel Sánchez-Velázquez, Alan Javier Hernández-Álvarez

**Affiliations:** 1 School of Food Science & Nutrition, University of Leeds, Leeds, United Kingdom; 2 National Alternative Protein Innovation Centre (NAPIC), Leeds, United Kingdom

**Keywords:** functional feed ingredients, gut homeostasis, intestinal barrier integrity, mucosal immunity, precision animal nutrition

## Abstract

Edible insects have emerged as promising sustainable feed ingredients capable of supporting both animal production and gastrointestinal health. Beyond their nutritional value, insect-derived meals contain bioactive compounds—including chitin, antimicrobial peptides, medium-chain fatty acids, micronutrients, and antioxidant molecules—that may modulate gut microbiota–host interactions in farmed monogastric animal species. This review critically examines current evidence on the mechanisms through which insect-based diets influence gastrointestinal physiology in livestock and aquaculture species. Available studies indicate that dietary insect inclusion can promote beneficial shifts in gut microbial communities, including increased bacterial diversity, enrichment of short-chain fatty acid-producing taxa, and reductions in selected opportunistic pathogens. However, these responses are not uniform across experimental models and depend on factors including insect species, processing method, dietary inclusion level, host species, age, and physiological status. Rather than acting through isolated mechanisms, insect-derived bioactive compounds function as an integrated nutritional matrix that simultaneously influences microbial metabolism, epithelial signaling, and immune regulation. Future standardized and multi-omics studies will be essential to identify the principal bioactive compounds responsible for these physiological effects and to optimize insect-derived functional ingredients for precision animal nutrition and more sustainable livestock and aquaculture production.

## 1 Introduction

The increasing global demand for animal-derived protein, together with the environmental and economic limitations associated with conventional feed ingredients, has intensified the search for sustainable alternatives in animal nutrition (Dicke, 2018; Sogari et al., 2019). Feed represents one of the highest production costs in livestock and aquaculture systems, particularly in monogastric species such as poultry, swine, and fish, where protein-rich ingredients including soybean meal and fishmeal remain essential dietary components (Kumar et al., 2017; Banhazi et al., 2012). However, the rising competition for these raw materials, coupled with concerns regarding land use, greenhouse gas emissions, marine resource depletion, and price volatility, has accelerated the development of alternative protein sources capable of supporting productive performance while reducing the environmental footprint of animal production systems (Makkar et al., 2014).

Among the emerging alternatives, insect-derived feed ingredients have gained substantial attention during the last decade. Insects such as the black soldier fly (BSF, *Hermetia illucens*) and the yellow mealworm (YM, *Tenebrio molitor*), have become particularly relevant because of their efficient bioconversion capacity, favorable amino acid profiles, lipid content, and ability to be reared on organic side streams (Van Huis and Gasco, 2023; Van Huis, 2020). Beyond their role as alternative protein sources, insect-derived meals contain a variety of functional compounds, including chitin, antimicrobial peptides (AMP), medium-chain fatty acids (MCFA), and other bioactive molecules, which may exert biological effects beyond conventional nutrition (Van der Fels‐Klerx et al., 2018; Van Huis, 2020).

Numerous studies conducted in poultry, swine, and aquaculture species have demonstrated that partial replacement of conventional protein sources with insect meals can maintain or, in some cases, improve productive parameters without compromising animal health (Veldkamp and Bosch, 2015; Gasco et al., 2019; Aslam et al., 2025). However, despite the growing body of evidence supporting the nutritional value of insects, a more mechanistic understanding of their physiological effects remains under development.

Over the last decade, increasing attention has been directed toward the gastrointestinal tract as a critical interface between dietary components, microbial communities, and host physiology (Kogut and Arsenault, 2016). The gastrointestinal system plays a central role not only in nutrient digestion and absorption but also in immune regulation, epithelial integrity, metabolic signaling, and disease resistance (Kogut and Arsenault, 2016; Oke et al., 2025; Chen et al., 2025). Within this context, the gut microbiota has emerged as one of the most influential biological systems affecting animal performance and health. The intestinal microbiome contributes to energy harvesting, fermentation of non-digestible substrates, production of short-chain fatty acids (SCFA), competitive exclusion of pathogens, and modulation of host immune responses (Zhang et al., 2025).

Insect-derived ingredients represent a particularly interesting dietary intervention because their biological composition suggests multiple pathways through which they may influence gut microbiota–host interactions (Abenaim and Conti, 2025). Chitin, a structural polysaccharide present in the insect exoskeleton, may act as a fermentable substrate for selected microbial taxa, potentially exerting prebiotic-like effects (Pascon et al., 2025). Likewise, insect lipids—especially lauric acid-rich fractions—have demonstrated antimicrobial properties that may influence microbial community structure (Aslam et al., 2025). Together, these components suggest that insect meals may function not only as nutrient sources but also as bioactive modulators of gastrointestinal physiology. Although these findings are promising, results remain inconsistent across studies due to differences in insect species, processing methods, inclusion levels, developmental stage of the insect, animal species, age, production system, and analytical methodologies.This heterogeneity has limited the establishment of clear mechanistic conclusions regarding how insect-derived feed ingredients influence gut microbiota–host interactions across food-producing vertebrates (Chen et al., 2025; Zhang et al., 2025; Rimoldi et al., 2019; Van Huis and Gasco, 2023). Moreover, most published reviews have focused predominantly on nutritional composition, digestibility, or productive performance, with comparatively less emphasis on microbiota-mediated physiological mechanisms. As a result, important questions remain unresolved regarding the extent to which insect-derived bioactive compounds contribute to microbial modulation, how these microbial shifts translate into host physiological responses, and whether such effects consistently improve gastrointestinal functionality and production outcomes.

Therefore, the objective of this review is to critically examine current evidence regarding the impact of edible insect-based feeds on gut microbiota–host interactions in monogastric livestock and aquaculture species. Particular emphasis will be placed on the functional roles of chitin, antimicrobial peptides, and lipid fractions as modulators of intestinal microbial ecology, mucosal integrity, immune responses, and gastrointestinal physiology. By integrating evidence across poultry, swine, and fish production systems, this review aims to identify common mechanistic patterns, highlight knowledge gaps, and propose future research directions for the rational development of functional insect-based feeds capable of supporting sustainable animal production.

## 2 Literature search and selection strategy

This review was developed as a narrative, mechanistically oriented review supported by a structured literature search and selection strategy. The purpose of the search was not to perform a systematic review or meta-analysis, but to identify and critically integrate the most relevant experimental evidence concerning the effects of edible insect-derived feed ingredients and their bioactive constituents on gastrointestinal physiology in monogastric livestock and aquaculture species. Accordingly, the review was not conducted under a PRISMA framework, and no attempt was made to estimate the proportion of all publications available in the field that was ultimately included. Instead, study selection was guided by relevance to the physiological and mechanistic scope of the manuscript.

Relevant literature was identified primarily through searches of Scopus, Web of Science, PubMed, and Google Scholar (**Figure 1**). Searches combined terms related to insect-derived feed ingredients and commonly studied insect species, including “insect meal”, “insect-based feed”, “black soldier fly”, “*Hermetia illucens*”, “yellow mealworm”, and “*Tenebrio molitor*”, with terms describing gastrointestinal and physiological outcomes, including “gut microbiota”, “intestinal microbiome”, “intestinal morphology”, “intestinal barrier”, “tight junction”, “mucin”, “mucosal immunity”, “immune response”, “oxidative stress”, “antioxidant”, “short-chain fatty acids”, “digestibility”, and “gut health”. Additional combinations incorporated specific functional components relevant to the scope of the review, such as “chitin”, “chitooligosaccharides”, “antimicrobial peptides”, “lauric acid”, “medium-chain fatty acids”, “insect lipids”, and “bioactive peptides”. Reference lists of particularly relevant primary articles and recent reviews were also examined to identify additional studies that could contribute mechanistic or historical context.

**Figure 1:**
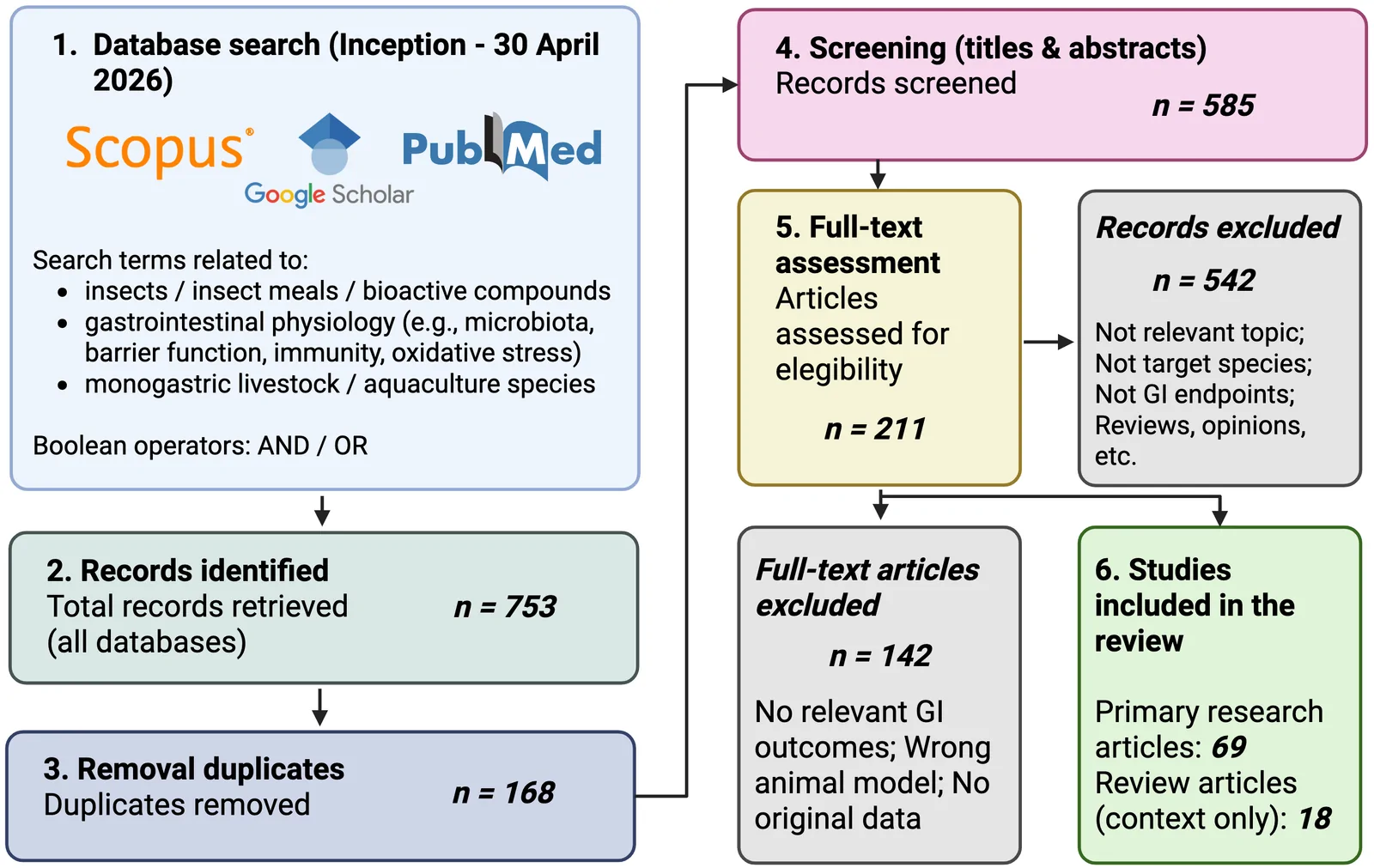
Overview of the literature search and study-selection strategy used in this narrative review.

Literature selection focused primarily on studies conducted in poultry, swine, fish, and crustacean production species. Original research articles were prioritized when they evaluated whole insect meals, processed insect ingredients, insect-derived fractions, or isolated bioactive components and reported outcomes directly relevant to gastrointestinal physiology. These outcomes included changes in gut microbial composition or diversity, microbial metabolites, intestinal morphology, mucin production, epithelial barrier-related markers, inflammatory or immune responses, oxidative status, pathogen resistance, and nutrient digestion when directly related to gastrointestinal function. *In vitro* or ex vivo investigations were considered when they provided mechanistic evidence that could help interpret findings from animal feeding studies, particularly for antimicrobial activity, microbial fermentation, or interactions between insect-derived compounds and intestinal cells.

Studies were considered particularly relevant when they provided sufficient information on the animal species or experimental model, insect ingredient or bioactive component, dietary inclusion or exposure conditions, and gastrointestinal outcome evaluated. Studies focusing exclusively on environmental sustainability, insect rearing performance, consumer acceptance, or productive performance without gastrointestinal or mechanistically relevant physiological endpoints were not prioritized for the core evidence synthesis. Research conducted in ruminants, companion animals, or humans was generally outside the principal scope of the review, except where mechanistic evidence was considered necessary to contextualize a biological pathway for which direct evidence in livestock or aquaculture species remains limited.

Primary research was preferentially used to support statements describing specific biological effects. Review articles were used mainly to establish broader nutritional or physiological context, identify relevant primary literature, and discuss areas in which evidence remains fragmented. Recent studies were preferentially considered when they provided new mechanistic information, while earlier studies were retained when they represented foundational evidence or remained particularly relevant to the interpretation of insect-derived feed ingredients.

The selected studies were critically compared according to host species, insect species or ingredient, ingredient processing, dietary inclusion or replacement level, gastrointestinal endpoint, and direction of the reported response. Particular attention was given to discrepancies among studies, including neutral or adverse outcomes and evidence of dose-dependent responses, rather than considering favorable effects as universally reproducible across animal models. This approach was used to distinguish experimentally demonstrated physiological responses from mechanisms that remain proposed or inferential. An overview of the literature identification and selection strategy used to construct this narrative review is presented in **Figure 1**.

## 3 Nutritional and biofunctional composition of edible insects

The nutritional relevance of edible insects in animal feeding systems is primarily associated with their high protein concentration, favorable amino acid profile, and lipid content (Bingqian et al., 2023). Beyond these conventional nutritional attributes, insect-derived ingredients contain structurally distinctive constituents—including chitin, antimicrobial peptides (AMPs), and characteristic lipid fractions rich in medium-chain fatty acids (MCFAs)—that may contribute additional biological functionality (Bingqian et al., 2023; Dicke, 2018). The presence and concentration of these compounds vary substantially according to insect species, developmental stage, rearing substrate, and processing conditions, making insect meals heterogeneous rather than compositionally interchangeable feed ingredients (Makkar et al., 2014).

Although the nutritional composition of insect meals varies considerably among species, developmental stage, rearing substrate, and processing conditions, several common patterns emerge (**Table 1**) (Hancz et al., 2024; Makkar et al., 2014; Siddiqui et al., 2025; Chobanova et al., 2023; Monteiro dos Santos et al., 2022; Motte et al., 2019; Ido et al., 2019). All insect meals are characterized by high protein concentrations, generally ranging from approximately 40% to 70% on a dry matter (DM) basis, positioning them as competitive alternatives to conventional protein sources such as soybean meal and fishmeal. Among the species most extensively studied, house cricket (HC, *Acheta domesticus*) exhibits one of the highest crude protein contents. Both species also provide essential amino acids such as lysine, methionine, threonine, and valine at concentrations (>40 g/100 g protein) compatible with the nutritional requirements of monogastric livestock and aquaculture species (Gasco et al., 2020; Ido et al., 2019; Monteiro dos Santos et al., 2022).

**Table 1:** Nutritional composition of selected insect meals with potential use as animal feed.

Scientific name	Common name	Form	Crude protein	Crude fat/ether extract	Ash	Fiber/chitin-related fraction	Nutritional relevance	Reference
*Hermetia illucens*	Black soldier fly (BSF)	Full-fat; defatted larvae meal	37–63% DM	7–39% DM	9–28% DM	5.3–16; affected by larval stage and processing	High-protein ingredient with relatively high lipid and mineral contents; defatting increases protein concentration and improves suitability for aquafeeds and monogastric diets	Makkar et al., 2014; Chobanova et al., 2023; Monteiro dos Santos et al., 2022
*Tenebrio molitor*	Yellow mealworm (YM)	Larvae meal	47–60% DM	31–43% DM	<5% DM	~5–8% DM; may increase with chitin fraction	Protein- and lipid-rich ingredient suitable for partial replacement of soybean meal or fishmeal; low calcium may require mineral balancing	Makkar et al., 2014; Motte et al., 2019; Ido et al., 2019
*Acheta domesticus*	House cricket (HC)	Cricket meal	63–70% DM	17–24% DM	5–6% DM	~10% DM ADF/chitin-associated fiber	High-protein insect meal with favorable essential amino acid profile; relevant for poultry, fish, and pet-food formulations.	Siddiqui et al., 2025; Gasco et al., 2020; Ido et al., 2019

Values are expressed as percentages in dry matter (% DM) basis.

However, crude protein concentration alone should not be interpreted as equivalent to nutritionally available protein. Conventional nitrogen-based estimates may overestimate true protein content because insects contain non-protein nitrogen, particularly within the chitinous exoskeleton (Boulos et al., 2020). Moreover, the chitin-containing matrix may restrict enzyme accessibility to proteins, making digestibility dependent on insect species, developmental stage, processing conditions, and dietary inclusion level (Bonomini et al., 2024). Consequently, digestible protein and amino acid availability are more informative indicators of the nutritional contribution of insect meals than crude protein concentration alone.

Meanwhile, YM and BSF, provide greater lipid fractions (31–43% DM and 7–39% DM, respectively), which may contribute additional energy but also influence the oxidative stability and health-promoting properties of feed formulations (Makkar et al., 2014). Conversely, defatted meals of black soldier fly larvae (BSFL) substantially increase protein concentration while reducing lipid-related technological limitations, making them particularly attractive for protein-enriched meals. In BSFL, in which lauric acid (C12:0) can constitute a substantial proportion of total fatty acids depending on rearing and processing conditions. Importantly, its antimicrobial potential has been evaluated experimentally rather than inferred solely from its chemical composition. Spranghers et al. (2018) reported that black soldier fly (BSF) fat containing 0.58 g C12:0/100 mL inhibited D-streptococci *in vitro*, while increasing the concentration to 0.87 g C12:0/100 mL resulted in an approximately 2-log reduction in bacterial counts. In piglets receiving BSF-based diets, a smaller but measurable reduction of approximately 0.5 log in intestinal D-streptococci was observed. These results provide direct evidence that the BSF lipid fraction can influence selected intestinal bacterial populations, although the substantially lower magnitude of the *in vivo* response illustrates the limitations of extrapolating antimicrobial activity measured *in vitro* directly to the gastrointestinal environment.

Comparable antimicrobial activity has been demonstrated for BSFL-derived lipid fractions in aquaculture-relevant bacterial models. Mohamed et al. (2021) reported dose-dependent inhibition of multidrug-resistant *Aeromonas hydrophila* and *A. salmonicida*, with minimum inhibitory concentrations of 0.09 and 0.38 mg mL^−1^, respectively. Although this evidence was obtained outside an animal feeding model, it supports a direct antimicrobial capacity of insect-derived lipid fractions against pathogens relevant to aquaculture and provides a mechanistic basis for the microbial responses subsequently observed in feeding studies.

Beyond protein supply, the lipid fraction of edible insects has attracted increasing attention because of its functional implications. In particular, BSFL contain high concentrations of lauric acid (C12:0), a MCFA recognized for its antimicrobial properties (Suryati et al., 2023). Lauric acid has been associated with inhibitory activity against Gram-positive bacteria, including several pathogenic species relevant in animal production systems (Chiengsorn et al., 2025). This suggests that insect lipids may contribute to modulation of intestinal microbial ecology through selective antimicrobial effects, potentially favoring the establishment of more stable microbial communities (Spranghers et al., 2018; Suryati et al., 2023).

One of the most distinctive structural components of insects is chitin, a β-(1→4)-linked polymer of N-acetyl-D-glucosamine that forms the exoskeleton (Moussian, 2019). Chitin content varies considerably across insect species and developmental stages but may represent between 5% and 17% of dry matter in commonly used insect meals (Sudwischer et al., 2025; Hasan et al., 2023; Nogales‐Mérida et al., 2019) (**Figure 2**). Traditionally considered an anti-nutritional factor because of its limited digestibility in monogastric animals, chitin is now increasingly recognized as a bioactive substrate with potential prebiotic-like properties (López-Santamarina et al., 2020). Several studies suggest that chitin and its derivatives, including chito-oligosaccharides, may selectively stimulate the growth of beneficial bacterial taxa, enhance SCFA production, and modulate intestinal immune responses (López-Santamarina et al., 2020; Borrelli et al., 2017).

**Figure 2:**
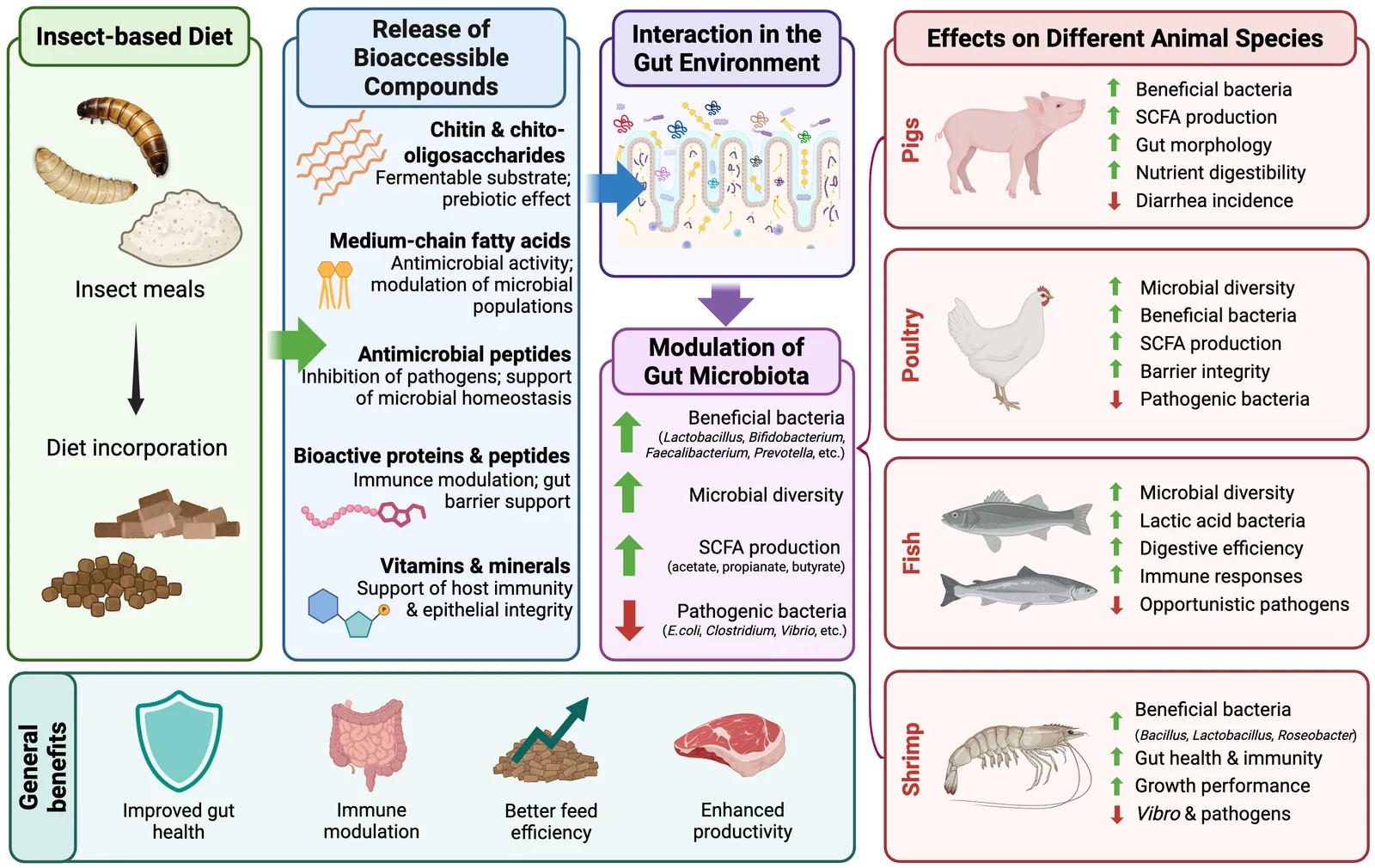
From bioaccessibility to bioactivity and gut-health promoting effects of insect meals on livestock and aquaculture species.

Chitin can also interact with the host immune system, although the nature of this response depends strongly on its physicochemical characteristics and the experimental context (Esteban et al., 2000; Cuesta et al., 2003). Primary studies using purified chitin have demonstrated that particle size and processing influence immune recognition, with different chitin fragments inducing distinct cytokine profiles through receptors including TLR-2, Dectin-1, and the mannose receptor (Komi et al., 2018). Experimental evidence from mammalian models indicates that these interactions can promote either pro-inflammatory or regulatory responses rather than uniformly beneficial immune stimulation. Therefore, activation of pattern-recognition pathways by chitin should not itself be interpreted as an indicator of improved gastrointestinal health.

Evidence from dietary studies in production-relevant species nevertheless indicates that chitin can modulate selected components of innate immunity. In gilthead seabream (*Sparus aurata*), Esteban et al. (2001) found that dietary chitin increased respiratory burst, natural haemolytic complement, and cytotoxic activities, whereas phagocytic and serum lysozyme activities were not enhanced. Similarly, dietary supplementation with chitin or chitosan in kelp grouper (*Epinephelus bruneus*) modified haematological and innate immune parameters and increased resistance to *Philasterides dicentrarchi* infection (Harikrishnan et al., 2012). Related dietary effects have also been reported in common carp exposed to *Aeromonas hydrophila* (Gopalakannan and Arul, 2006). Collectively, these studies support an immunomodulatory rather than universally immunostimulatory role for dietary chitin (Mousavi et al., 2020; Abenaim and Conti, 2025). However, these experiments generally evaluated purified chitin or chitosan that was not specifically isolated from edible insects. Direct evidence demonstrating that insect-derived dietary chitin itself activates defined pattern-recognition receptor pathways in livestock or aquaculture species therefore remains limited.

Consistent with this mechanistic possibility, feeding studies using chitin-containing insect meals have reported modulation of immune-related endpoints. For example, dietary BSFL meal in pearl gentian grouper was associated with changes in NF-κB-, TLR-, MyD88-, and IL-10-related responses, while other insect-meal studies in fish and poultry have reported alterations in lysozyme activity, cytokine expression, and lymphocyte populations (Komi et al., 2018).

Partially defatted BSF meal exhibited significantly greater apparent metabolizable energy (AME = 16.25 MJ/kg DM; AMEn=14.87 MJ/kg DM; *P* < 0.05) than highly defatted meal, while amino acid apparent ileal digestibility coefficients ranged from 0.44 to 0.92 and 0.45 to 0.99 for partially and highly defatted meals, respectively. Overall amino acid digestibility remained high (77–80%), supporting defatted BSF meal as an excellent source of digestible protein and metabolizable energy for poultry. Nevertheless, because whole insect meals contain several potentially bioactive constituents, including antimicrobial peptides, fatty acids, and other compounds, these responses cannot be attributed specifically to chitin without studies using isolated insect-derived chitin or appropriate fractionated controls.

Antimicrobial peptides (AMP) represent another group of insect-derived bioactive compounds with potential relevance to gastrointestinal physiology. Insects produce several AMP families, including defensins, cecropins, attacins, and diptericins, as part of their innate immune defense (Patyra and Kwiatek, 2023; Wu et al., 2018). Experimental studies using isolated insect-derived peptides have demonstrated direct antimicrobial activity against selected bacterial and fungal targets, supporting a plausible role for these compounds in modulating microbial populations. For example, Fu et al. (2009) purified an antifungal peptide from the haemolymph of *Musca domestica* and demonstrated inhibitory activity against fungal targets, providing direct evidence of the antimicrobial potential of insect-derived peptides.

However, the extent to which these effects are reproduced following dietary inclusion of whole insect meals remains less clear. Feeding studies with insect-based ingredients frequently report changes in intestinal microbial composition, pathogen abundance, or immune-related markers, but these responses cannot be attributed specifically to AMPs because whole insect meals also provide chitin, fatty acids, proteins, and other bioactive compounds. In addition, peptide stability during processing and gastrointestinal digestion may influence their biological availability. Therefore, while isolated insect AMPs clearly possess antimicrobial activity, their specific contribution to microbiota modulation and host defense following dietary insect inclusion in livestock and aquaculture species remains to be established experimentally (Alhasyani et al., 2025; Xia et al., 2021; Sibinga, 2022).

Insect meals also provide minor constituents whose concentrations depend strongly on species, rearing substrate, and processing. Micronutrients also contribute to the functional profile of edible insects. Insect meals contain significant concentrations of minerals (ashes <5–6% DM, **Table 1**)–such as iron, zinc, selenium, and phosphorus–, B-complex vitamins, and antioxidant compounds, depending on species and rearing substrate (Astuti and Komalasari, 2020). These nutrients may indirectly support gut physiology through their roles in epithelial turnover, antioxidant defense, and immune competence (Bingqian et al., 2024). Because the gastrointestinal relevance of these compounds is less well established and, in several cases, remains mechanistically distinct from the major structural components described above, their available experimental evidence is discussed separately in Section 5.

Importantly, antimicrobial activity demonstrated with isolated insect peptides should not be assumed to occur to the same extent following consumption of whole insect meals, because peptide concentration, gastrointestinal proteolysis, and feed-processing conditions may substantially affect their bioavailability (Melgar-Lalanne et al., 2019; Hernández-Álvarez et al., 2021). Understanding how these mechanisms interact under different production contexts is essential for interpreting the reported effects of insect-based diets on gut microbiota–host interactions in food-producing animals.

## 4 Gut microbiota modulation by insect-based diets

Dietary inclusion of insect-derived feed ingredients has been associated with measurable changes in the composition, diversity, and metabolic activity of the gut microbiota in food-producing animals (Biasato et al., 2024). One proposed mechanism through which these ingredients may influence microbial communities is the fermentation of structural polysaccharides such as chitin (**Figure 2**). Although chitin is poorly digested by endogenous enzymes in many monogastric animals, it may serve as a substrate for microbial populations with chitinolytic capacity (Veldkamp et al., 2022). This fermentation may contribute to shifts in microbial community structure and to the production of metabolites such as short-chain fatty acids, including acetate, propionate, and butyrate. However, the relative contribution of chitin fermentation to the overall microbiota response remains uncertain and is likely to vary according to host species, insect ingredient, processing method, and dietary inclusion level (Elnesr et al., 2022**;** Lokman et al., 2019**).**

### 4.1 Poultry

Research in poultry has demonstrated that insect-derived ingredients can beneficially modulate the gut microbiota (**Figure 3**). For example, dietary replacement of fishmeal with BSFL meal maintained gut microbial communities comparable to conventional diets while increasing the abundance of beneficial lactic acid-producing bacteria (Ndotono et al., 2022). In layer pullets, Ndotono et al. (2022) evaluated the progressive replacement of dietary fishmeal with BSFL meal, ranging from 0% replacement in the control diet to complete fishmeal replacement with BSFL. Across treatments, Firmicutes was the dominant phylum, followed by Bacteroidetes, Proteobacteria, and Actinobacteria (~2% each), while *Lactobacillus* predominated at the genus level (93%). Overall, BSFL-fed birds maintained a microbial community broadly comparable to that of the fishmeal-fed control at both phylum and genus levels, although the relative abundance of several potentially beneficial taxa, including *Lactobacillus*, Bacteroides, *Blautia*, and *Enterococcus*, increased with increasing BSFL inclusion. The group receiving complete replacement of fishmeal with BSFL showed the highest alpha diversity and species richness; however, beta-diversity analysis revealed only limited differences in overall community structure among dietary treatments (PERMANOVA, p > 0.05). These findings suggest that BSFL inclusion primarily modified the relative abundance and richness of selected microbial populations rather than producing a wholesale restructuring of the intestinal microbiota.

**Figure 3:**
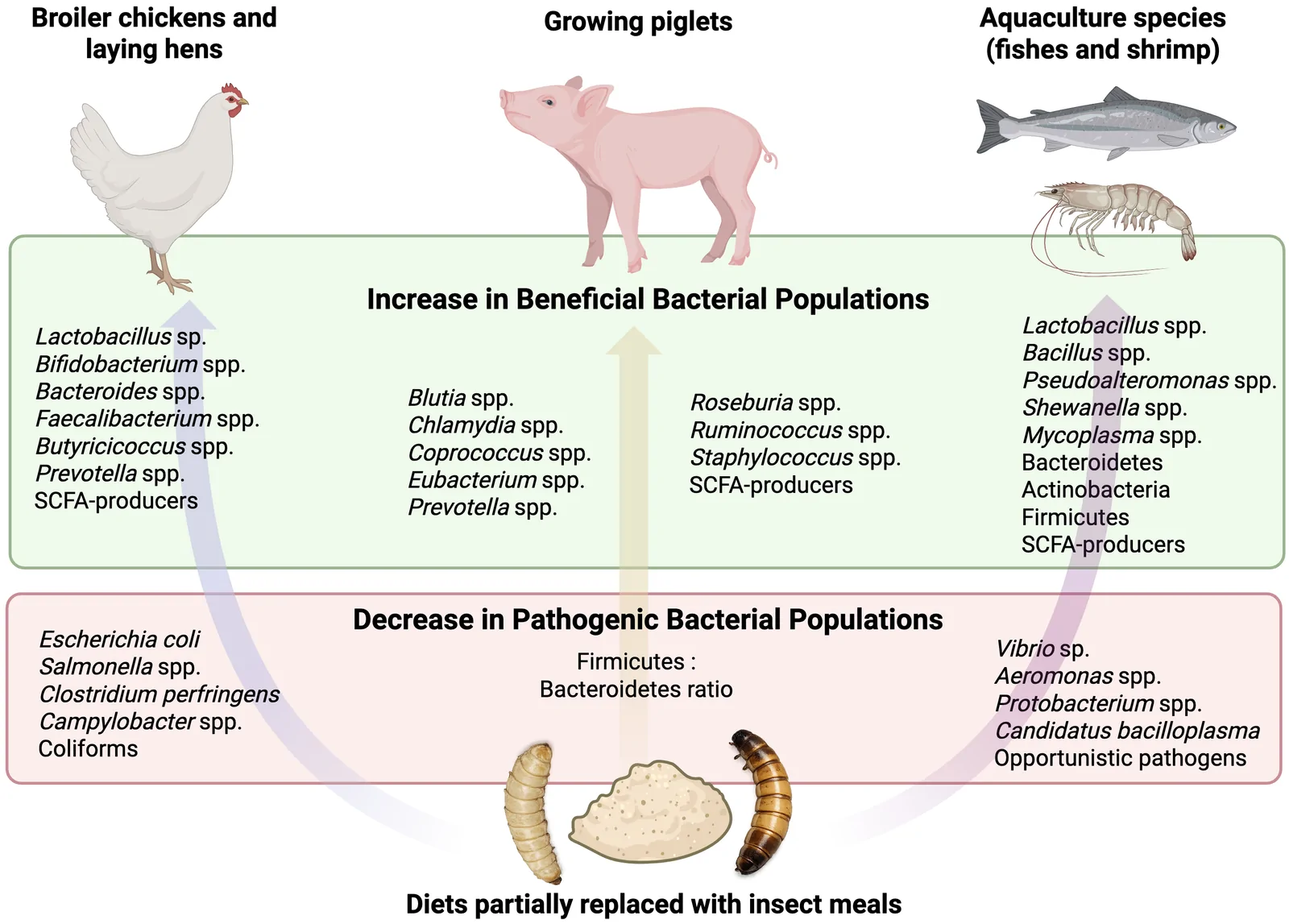
Modulating effects of insect meal on the beneficial and pathogenic bacterial population of livestock and aquaculture animal species. The responses illustrated are based on representative primary studies discussed throughout Section 4. Key sources informing the figure include Biasato et al. (2018, 2020, 2024), Bruni et al. (2018); Chen et al. (2021); Elnesr et al. (2022); Guevarra et al. (2019); He et al. (2022); Lokman et al. (2019); Magalhães et al. (2017); Mohamed et al. (2021); Ndotono et al. (2022); Rimoldi et al. (2019); Schiavone et al. (2017); Terova et al. (2019); Veldkamp et al. (2022), and Wang et al. (2025).

Biasato et al. (2018) demonstrated that dietary inclusion of 7.5% YM meal positively modulated the cecal microbiota of free-range chickens without adversely affecting intestinal morphology or mucin composition. Birds receiving the YM-based diet exhibited significantly greater microbial alpha diversity (Shannon index, *P* < 0.05) and beta diversity (Adonis and ANOSIM, *P* < 0.001) than controls. The dietary treatment also increased the Firmicutes: Bacteroidetes ratio (*FDR* < 0.05) and promoted higher relative abundances of bacterial genera commonly associated with gut homeostasis, including *Clostridium*, *Oscillospira*, *Ruminococcus*, *Coprococcus*, and *Sutterella* (*FDR* < 0.05), while reducing the abundance of *Bacteroides*. Importantly, these microbial shifts occurred without significant changes in villus height, crypt depth, villus height-to-crypt depth ratio, or intestinal mucin composition, suggesting that YM meal can beneficially modulate the gut microbial ecosystem while maintaining intestinal structural integrity.

Similar findings have been reported with BSFL meal, where dietary supplementation altered microbial composition while maintaining productive performance and gut integrity (Schiavone et al., 2017). Collectively, poultry studies indicate that dietary insect ingredients can modify the intestinal microbial ecosystem, although the magnitude and direction of these responses vary according to insect species, inclusion level, ingredient processing, and experimental conditions. Reported changes include alterations in microbial diversity and in the relative abundance of bacterial taxa associated with carbohydrate fermentation and intestinal homeostasis. Importantly, these responses do not necessarily involve extensive restructuring of the microbial community, as some studies have reported relatively conserved dominant taxa alongside changes in the abundance of specific bacterial groups.

### 4.2 Swine

In swine, particularly during the post-weaning period, modulation of gut microbiota is especially relevant because early-life microbial instability often predisposes piglets to gastrointestinal disorders, inflammation, and reduced feed efficiency (Guevarra et al., 2019). Primary feeding studies in swine indicate that dietary insect inclusion can modify the intestinal microbial ecosystem, although the responses depend on the insect ingredient and dietary inclusion level (Biasato et al., 2020; Meyer et al., 2020; Spranghers et al., 2018). In weaned piglets, Biasato et al. (2020) evaluated diets containing 5% or 10% BSFL meal and reported increased cecal microbial beta diversity (P < 0.001), together with greater relative abundance of several genera associated with fiber fermentation and intestinal homeostasis, including *Blautia*, *Coprococcus*, *Eubacterium*, *Prevotella*, *Roseburia*, *Ruminococcus*, and unclassified Ruminococcaceae (FDR < 0.05). These microbial changes were accompanied by a higher proportion of neutral mucins in the small intestine (P < 0.05), while mature mucin secretory architecture was preserved and no intestinal inflammation was observed (**Figure 3**). These microbial changes were accompanied by a higher proportion of neutral mucins in the small intestine (*P* < 0.05), preservation of mature mucin secretory architecture, and the absence of intestinal inflammation, suggesting that BSFL meal supports both microbial and mucosal components of the intestinal barrier.

Comparable observations have also been reported following dietary inclusion of YM meal in growing pigs (Meyer et al., 2020). Replacement of soybean meal with 5% or 10% YM meal induced significant shifts in the cecal microbial community, characterized by a reduction in the relative abundance of *Bacteroidetes* (*P* < 0.05) and a tendency toward increased *Firmicutes* abundance and a higher Firmicutes ratio (*P* < 0.1). Although total SCFA concentrations remained unchanged, both insect-supplemented diets significantly increased the concentrations of branched-chain fatty acids (*P* < 0.05), indicating alterations in microbial fermentation activity. These findings demonstrate that insect-derived ingredients not only reshape microbial community composition but also influence microbial metabolic function, providing further evidence that insect meals can modulate the intestinal ecosystem through complementary effects on both microbial structure and fermentation dynamics (Meyer et al., 2020).

Additional evidence suggests that the beneficial effects of BSF-derived ingredients may also be mediated by their lipid fraction, particularly lauric acid (C12:0), which possesses well-documented antimicrobial activity against Gram-positive bacteria (Spranghers et al., 2018). *In vitro*, BSF fat containing 0.58 g C12:0/100 mL inhibited the growth of *D*-streptococci, whereas supplementation with 0.87 g C12:0/100 mL resulted in approximately a 2-log reduction in bacterial counts. Similar, although less pronounced, effects were observed *in vivo*, where piglets receiving BSF-based diets exhibited approximately 0.5-log reductions in intestinal *D*-streptococci populations. Importantly, replacement of soybean products with BSF meal did not impair animal performance or overall protein digestibility (77–78%), while diets containing 4% full-fat or defatted BSF meal improved ileal protein digestibility to approximately 73.3%, compared with 69.7% in the control diet. These findings indicate that, beyond chitin-mediated prebiotic effects, insect-derived lipids may contribute to gut health by selectively suppressing undesirable bacterial populations while maintaining nutrient utilization efficiency.

### 4.3 Aquaculture species

In aquaculture species, the interaction between insect-based diets and the gut microbiota has also received increasing attention. Fish species such as rainbow trout (*Oncorhynchus mykiss*), Nile tilapia (*Oreochromis niloticus*), and European seabass (*Dicentrarchus labrax*) have demonstrated beneficial physiological responses following dietary inclusion of BSFL meal (**Figure 3**). In rainbow trout, partial replacement of fishmeal with 10%–30% BSFL meal significantly increased gut microbial richness and diversity while promoting the abundance of beneficial lactic acid- and butyrate-producing bacteria, effects that were attributed primarily to the fermentable chitin fraction of the insect meal (Terova et al., 2019).

Similarly, juvenile Nile tilapia receiving diets in which 60% of soybean meal was replaced by defatted BSFL meal exhibited significantly higher survival rates following *Aeromonas veronii* infection, reduced intestinal inflammation, increased goblet cell numbers, enhanced mucin 2 (*muc2*) expression, and dose-dependent stimulation of mucin production and secretion (*P* < 0.05). These responses were accompanied by alterations in gut microbial composition and increased intestinal concentrations of L-tyrosine, a metabolite that subsequently upregulated *muc2* and *spdef* expression in intestinal epithelial cells, providing mechanistic evidence linking insect-derived ingredients to improved mucosal barrier function (Wang et al., 2025).

Complementary findings have been reported in European seabass, where replacement of up to 45% of fishmeal (19.5% BSFL meal; equivalent to 22.5% of total dietary protein) maintained growth performance, feed utilization, and overall nutrient digestibility while increasing the apparent digestibility coefficients of arginine, histidine, and valine (Magalhães et al., 2017).

Reported microbial responses across aquaculture species indicate that insect-derived ingredients promote a healthier intestinal ecosystem. In rainbow trout, partial replacement of fishmeal with BSFL meal increased bacterial richness and diversity while enriching lactic acid- and butyrate-producing bacteria, effects that have been largely attributed to the fermentable chitin fraction of the insect meal (Terova et al., 2019; Rimoldi et al., 2019). Similar increases in microbial biodiversity and marked shifts in bacterial community structure were reported when 25–50% of fishmeal was replaced with partially defatted BSFL meal, with significant changes in both mucosa-associated and digesta-associated bacterial communities (ANOSIM *P* < 0.05 and *P* < 0.01, respectively) (Bruni et al., 2018).

Comparable responses have also been documented in crustacean aquaculture. In Pacific white shrimp (*Litopenaeus vannamei*), dietary replacement of 10–20% of fishmeal protein with BSFL meal reduced the relative abundance of opportunistic genera such as *Vibrio*, *Photobacterium*, and *Candidatus* Bacilloplasma, while increasing potentially beneficial bacteria including *Bacillus* and *Pseudoalteromonas*. These microbial changes were associated with improved survival following *Vibrio parahaemolyticus* challenge, whereas excessive inclusion levels (30%) negatively affected intestinal morphology and immune-related gene expression, highlighting the importance of optimizing dietary inclusion rates (Chen et al., 2021). Likewise, partial replacement of commercial feeds with fresh BSFL increased intestinal bacterial richness and diversity, promoted potentially probiotic genera such as *Motilimonas*, *Shimia*, *Pseudoalteromonas*, and *Shewanella*, reduced *Vibrio* abundance, and enhanced immune-related enzyme activities, including total nitric oxide synthase, alkaline phosphatase, and acid phosphatase (He et al., 2022). Beyond dietary supplementation, bioactive lipid fractions isolated from BSFL have also demonstrated potent dose-dependent antibacterial activity against multidrug-resistant *Aeromonas hydrophila* and *Aeromonas salmonicida*, with minimum inhibitory concentrations as low as 0.09 and 0.38 mg mL⁻¹, respectively, suggesting that insect-derived lipids may contribute directly to pathogen control in aquaculture systems (Mohamed et al., 2021).

Taken together, studies across poultry, swine, fish, and crustacean species indicate that dietary insect-derived ingredients can modify the intestinal microbial ecosystem, although the magnitude and direction of these responses vary substantially among host species, insect ingredients, processing conditions, and inclusion levels. Across animal models, recurrent responses include changes in microbial diversity and relative abundance of taxa associated with carbohydrate fermentation and intestinal homeostasis, together with reductions in selected opportunistic bacterial populations. However, these effects are not uniformly observed and do not necessarily involve extensive restructuring of the entire microbial community. Several studies also link microbial changes with alterations in fermentation metabolites, mucin dynamics, epithelial barrier-related responses, or resistance to enteric pathogens, suggesting functional interactions between microbial modulation and host gastrointestinal physiology. Importantly, the available evidence also indicates dose-dependent and ingredient-specific responses, with high inclusion levels occasionally associated with adverse intestinal outcomes. Overall, current findings support microbiota modulation as an important component of the gastrointestinal response to insect-based diets, while also emphasizing that no single microbial signature can yet be considered universal across monogastric livestock and aquaculture species.

## 5 Insect-derived minor bioactive compounds as modulators of gastrointestinal physiology

### 5.1 Micronutrients

Although proteins, chitin, and lipid fractions represent the major functional components of edible insects, insect-derived feed ingredients also provide a diverse array of micronutrients and minor bioactive compounds that may contribute to gastrointestinal physiology. These constituents include essential trace minerals, vitamins, phospholipids, sterols, antioxidant pigments, and low-molecular-weight metabolites whose biological activity has received comparatively less attention but may complement the effects of the major bioactive fractions. The concentration of micronutrients varies considerably among insect species and is strongly influenced by the rearing substrate, developmental stage, and processing conditions (Mabelebele et al., 2023). Although these nutrients primarily support systemic metabolic functions, they also play essential roles in maintaining intestinal homeostasis.

Insect meals contain trace minerals, including zinc, selenium, copper, and manganese; however, direct experimental evidence demonstrating that the mineral fraction of insect-based feeds independently modulates gastrointestinal physiology remains limited. An emerging application is the use of insects as carriers of supplemented trace elements. For example, Mekapothula et al. (2026) evaluated the biofortification of BSFL with zinc, selenium, manganese, and copper nanoparticles and demonstrated increased accumulation of these elements in the insect biomass. Notably, zinc accumulated in BSFL showed greater bioavailability in broiler chickens than conventional zinc oxide supplementation. This study provides preliminary evidence that insect biomass may serve as a vehicle for delivering selected trace elements, although further feeding studies are required before this functionality can be generalized across minerals, insect species, or animal production systems.

From an applied perspective, insect meals have demonstrated productive and intestinal benefits comparable to those achieved with conventional zinc bacitracin supplementation. Diets containing *Macrotermes subhyalinus* meal improved cecal bacterial composition, crude protein digestibility, feed conversion ratio, and growth performance in broiler chickens without adverse effects on metabolic health (Malematja et al., 2026). Although these responses cannot be attributed solely to their mineral content, they reinforce the concept that insect meals constitute multifunctional feed ingredients whose physiological effects likely result from synergistic interactions among proteins, bioactive compounds, and micronutrients. Nevertheless, studies specifically designed to elucidate the gastrointestinal roles of insect-derived trace minerals in swine, poultry, and aquaculture species remain scarce, representing an important avenue for future research.

### 5.2 Minor bioactive constituents

Insect-derived lipid fractions contain minor constituents such as phospholipids and sterols in addition to their predominant fatty acid components. Although these molecules are known to participate in membrane organization and cellular signaling, direct evidence demonstrating that phospholipids or sterols specifically derived from edible insects modulate intestinal epithelial physiology in livestock or aquaculture species remains scarce (Siddiqui et al., 2025; Lv et al., 2026; Wijesekara and Xu, 2024). Consequently, their proposed contribution to gastrointestinal function should currently be regarded as a mechanistic hypothesis rather than an effect demonstrated *in vivo* through insect-based feeding studies.

Another emerging area of research concerns the contribution of insect-derived antioxidant compounds and redox-active nutrients to gastrointestinal homeostasis. Edible insects contain a diverse array of accessory bioactive molecules, including melanin, carotenoid-like pigments, phenolic compounds acquired from the rearing substrate, antioxidant peptides, and trace minerals involved in cellular redox regulation. Although these compounds generally occur at lower concentrations than those found in plant-derived ingredients, increasing evidence suggests that insect-based diets enhance antioxidant defenses through the synergistic action of their complex nutritional matrix rather than through a single bioactive constituent.

Experimental evidence from both terrestrial and aquatic production species supports this concept. In broiler chickens exposed to heat stress, replacing 5% of the diet with YM, BSF, or *Zophobas morio* larvae improved productive performance, with birds receiving BSF exhibiting the highest body weight, while YM supplementation significantly reduced hepatic malondialdehyde (MDA), a marker of lipid peroxidation, and BSF increased total phenolic content in breast muscle (Stelios et al., 2024). Although total antioxidant capacity (TAC) and DPPH radical scavenging activity remained unchanged, the reduction in hepatic MDA observed with YM suggests that this dietary intervention may attenuate lipid peroxidation under heat-stress conditions. However, the absence of corresponding changes in global antioxidant indices indicates that this response should not be interpreted as a generalized enhancement of antioxidant capacity (Stelios et al., 2024).

Comparable responses have been reported in aquaculture. Juvenile mirror carp fed diets containing BSF pulp at inclusion levels of 43.7, 87.3, 131, and 174.7 g/kg showed significantly increased serum catalase activity together with reduced MDA concentrations. Importantly, BSF pulp inclusion levels below 131 g/kg did not compromise growth performance or intestinal health, whereas the highest inclusion level (174.7 g/kg) resulted in reduced villus height, villus area, and muscular layer thickness, suggesting that antioxidant benefits may not always parallel improvements in intestinal morphology (Xu et al., 2020). Likewise, supplementation of soybean-based diets with 8% insect meal improved growth performance and restored selected fatty acids in juvenile rainbow trout, whereas increasing inclusion to 16% did not confer additional benefits but was associated with reduced glutathione peroxidase (GPX) activity, indicating that antioxidant responses may be dose dependent rather than linearly proportional to insect meal inclusion (Hossain et al., 2021).

The mechanisms underlying the antioxidant responses observed following insect meal inclusion remain incompletely resolved and are likely multifactorial. Insect meals contain several constituents with established or proposed roles in redox regulation, including selenium, sulfur-containing amino acids, antioxidant peptides, vitamins, and other redox-active compounds (Tobolska et al., 2026). However, feeding studies generally evaluate whole insect ingredients and therefore do not allow the contribution of individual constituents, or potential synergistic interactions among them, to be determined independently. Consequently, the antioxidant responses reported *in vivo* should currently be interpreted as effects associated with the complex insect nutritional matrix rather than as evidence of a demonstrated coordinated mechanism among specific bioactive compounds.

Together, these findings support the view that the antioxidant capacity of insect-derived feed ingredients should be regarded as an emergent property of their integrated nutritional matrix rather than the consequence of isolated compounds (Bingqian et al., 2024). Future research combining metabolomics, redox biology, and gastrointestinal physiology will be essential to identify the principal antioxidant metabolites released during digestion, determine their bioaccessibility, and clarify how they interact with the gut microbiota and intestinal epithelium to enhance host resilience across livestock and aquaculture species.

### 5.3 Integrated physiological responses

Insect-based feeds should therefore be regarded as complex nutritional matrices whose effects on gastrointestinal physiology may result from interactions among multiple nutritional and bioactive constituents rather than from the action of a single compound. Among the gastrointestinal responses investigated following dietary inclusion of insect-derived ingredients, changes in intestinal barrier-related markers have received increasing attention. Some studies suggest that insect-based diets may enhance the expression of tight junction proteins, including occludin, claudin-1, claudin-3, and ZO-1 (Colombino, 2023; Li et al., 2024). These structural proteins regulate paracellular permeability and contribute to epithelial defense against luminal pathogens and toxins. However, the mechanisms responsible for their modulation following insect-based feeding remain incompletely established. Microbial metabolites such as butyrate, together with chitin-derived compounds, antimicrobial peptides, and other constituents of the insect matrix, represent plausible contributors, but their individual roles have not been clearly resolved in livestock and aquaculture species.

A second integrated response involves reinforcement of the intestinal mucus barrier, which constitutes the first line of defense separating luminal microorganisms from the intestinal epithelium. This protective layer not only limits pathogen adhesion and epithelial invasion but also, provides a favorable ecological niche for commensal microorganisms involved in maintaining intestinal homeostasis. Although evidence regarding the direct regulation of mucin production by insect-derived ingredients remains limited, several recent studies suggest that insect-based diets can positively influence components of the mucosal barrier.

For example, dietary inclusion of insect proteins has been associated with modulation of genes involved in mucus production and epithelial integrity. Broiler chickens receiving grasshopper (*Dociostaurus maroccanus*) meal at an 8% dietary inclusion exhibited significantly higher *MUC2* gene expression compared with birds fed BSFL diets, whereas *CLDN1*, a key tight junction protein, was significantly upregulated in BSFL-fed groups. Moreover, diets containing 24% BSFL improved body weight gain and ileal protein digestibility, while 24% grasshopper meal significantly reduced cecal *Escherichia coli* populations, indicating that insect-derived proteins may simultaneously enhance epithelial function and microbial homeostasis (Mohassessi et al., 2025).

Since butyrate is recognized as a major inducer of goblet cell differentiation and mucin synthesis, these observations support the hypothesis that insect-derived ingredients may strengthen the mucus barrier through microbiota-mediated mechanisms rather than solely through direct stimulation of mucin gene expression (Dalmoro et al., 2025). Additional evidence indicates that insect-derived diets may reinforce mucus barrier functionality indirectly through microbial metabolism. Under an experimental *Eimeria* spp. and *Clostridium perfringens* challenge, dietary inclusion of 5% BSFL meal increased cecal concentrations of acetate and butyrate while reducing intestinal IL-6 expression, without adversely affecting intestinal histomorphology (Dalmoro et al., 2025). Similarly, laying hens receiving expanded BSFL meal exhibited a significant reduction in TNF-α expression at lower dietary inclusion levels (3–6% EP-BSF), coinciding with increased serum estradiol concentrations and enhanced expression of the gap junction proteins GJA1 and GJA4, indicating that insect-derived ingredients may also influence immune regulation through endocrine and epithelial signaling pathways (Tajudeen et al., 2025).

Nevertheless, not all inflammatory mediators respond uniformly to insect supplementation. Colombino et al. (2021) reported that dietary supplementation with live BSF or YM larvae at 5% of the expected daily feed intake did not significantly alter jejunal expression of IL-4, IL-6, TNF-α, IFN-γ, or MUC2 in broiler chickens, although IL-2 expression was significantly reduced in birds receiving YM. Together with the observed enrichment of SCFA-producing bacterial taxa, these findings suggest that improvements in intestinal homeostasis may occur without major transcriptional changes in classical inflammatory cytokines, reinforcing the complexity of microbiota–host interactions.

Current evidence therefore supports the concept that insect-derived diets promote immune regulation rather than generalized immunosuppression, which is consistent with broader gastrointestinal research demonstrating that nutritional interventions capable of maintaining intestinal homeostasis typically reduce the expression of pro-inflammatory mediators such as TNF-α, IL-8, IL-18, and IFN-γ, while preserving or enhancing anti-inflammatory pathways involving IL-10 (Rodríguez-González et al., 2024). Although direct evidence for increased IL-10 or TGF-β expression following insect meal supplementation remains limited, the combined reduction of inflammatory cytokines, increased production of microbial SCFA, and preservation of epithelial integrity strongly suggest that insect-derived bioactive compounds contribute to maintaining immune equilibrium within the intestinal mucosa rather than eliciting excessive inflammatory activation.

The comparative evidence summarized in **Table 2** further illustrates that gastrointestinal responses to insect-derived feed ingredients are strongly dependent on inclusion level, host species, and the physiological endpoint evaluated. In poultry, for example, inclusion of 7.5% YM meal significantly increased cecal microbial α-diversity (Shannon index, *P* < 0.05) and β-diversity (Adonis and ANOSIM, *P* < 0.001), while also increasing the relative abundance of several genera associated with gut homeostasis, including *Clostridium*, *Oscillospira*, *Ruminococcus*, *Coprococcus*, and *Sutterella* (FDR<0.05). However, these microbial responses occurred without significant changes in villus height, crypt depth, villus height-to-crypt depth ratio, or intestinal mucin composition, indicating that changes in microbial ecology do not necessarily translate into simultaneous structural remodeling of the intestinal mucosa. Likewise, in weaned piglets, dietary inclusion of 5% or 10% BSFL meal significantly increased cecal microbial beta diversity (*P* < 0.001) and enriched *Blautia*, *Coprococcus*, *Eubacterium*, *Prevotella*, *Roseburia*, *Ruminococcus*, and *Ruminococcaceae* (FDR<0.05). These changes were accompanied by a higher proportion of neutral mucins in the small intestine (*P* < 0.05), preservation of mature mucin secretory architecture, and absence of intestinal inflammation, providing evidence that, under some conditions, microbiota modulation and mucosal responses can occur concurrently.

**Table 2:** Representative studies evaluating gastrointestinal responses to insect-derived feed ingredients in monogastric livestock and aquaculture species.

Animal species	Insect ingredient	Inclusion/replacement level	Ingredient form/processing	Main gastrointestinal endpoints	Main findings	Reference
Poultry	Yellow mealworm	7.5%	Meal	Microbiota; morphology; mucin	↑ α/β diversity; no adverse morphology/mucin changes	Biasato et al., 2018
Broilers	Black soldier fly larvae	5% under challenge	Whole insects	SCFA/cytokines/histomorphology	↑ acetate and butyrate, ↓ IL-6; no adverse histomorphology	Dalmoro et al., 2025.
Broilers	Black soldier fly larvae/grasshopper	Variable inclusion		muc2/CLDN1/digestibility/microbiota	ingredient-dependent responses	Mohassessi et al., 2025.
Piglets	Black soldier fly larvae	5/10%	Meal	Microbiota; mucin	↑ selected fermentative taxa; preservation of mucosal architecture	Biasato et al., 2020
Pigs	Yellow mealworm	5/10%	Meal	Microbiota/fermentation	Altered microbial community and ↑ BCFA; unchanged total SCFA	Meyer et al., 2020
Rainbow trout	Black soldier fly larvae	10–30%	Meal	Microbiota	↑ richness/diversity and selected lactic acid/butyrate-producing bacteria	Terova et al., 2019
Nile tilapia	Defatted black soldier fly larvae	60% soybean-meal replacement		Microbiota/barrier/infection response	↑ survival, goblet cells and muc2; ↓ inflammation	Wang et al., 2025
Mirror carp	Black soldier fly pulp	43.7–174.7 g/kg	Pulp	Antioxidant/morphology	Improved selected antioxidant endpoints at moderate levels; impaired morphology at highest level	Xu et al., 2020.
Pacific white shrimp	Black soldier fly larvae	10–30% fishmeal protein replacement		Microbiota/morphology/immunity	Favorable responses at 10–20%, adverse intestinal responses at 30%	Chen et al., 2021

The effects of insect meal inclusion on microbial metabolism are similarly heterogeneous. In growing pigs, replacement of soybean meal with 5% or 10% YM significantly reduced the relative abundance of Bacteroidetes (*P* < 0.05) and tended to increase Firmicutes abundance and the Firmicutes: Bacteroidetes ratio (*P* < 0.1). Nevertheless, total short-chain fatty acid concentrations remained unchanged, whereas branched-chain fatty acids increased significantly (*P* < 0.05). This distinction is relevant because it demonstrates that taxonomic shifts do not necessarily predict increased production of metabolites conventionally associated with intestinal health. Moreover, the lipid fraction of BSF may contribute additional antimicrobial activity. *In vitro*, BSF fat containing 0.58 g C12:0/100 mL inhibited D-streptococci, while 0.87 g C12:0/100 mL produced an approximately 2-log reduction in bacterial counts; *in vivo*, piglets receiving BSF-based diets showed a more moderate reduction of approximately 0.5 log. At the same time, diets containing 4% full-fat or defatted BSF improved ileal protein digestibility to approximately 73.3%, compared with 69.7% in the control diet, while overall protein digestibility remained within 77–78%. These observations suggest that microbial, antimicrobial, and digestive responses may coexist but differ substantially in magnitude between experimental models.

Comparable dose-dependent patterns are evident in aquaculture species. In rainbow trout, partial substitution of fishmeal with BSFL meal increased bacterial richness and diversity and enriched lactic acid- and butyrate-producing bacteria, whereas replacement of 25–50% of fishmeal with partially defatted BSFL meal significantly altered both mucosa-associated (ANOSIM *P* < 0.05) and digesta-associated (*P* < 0.01) microbial communities. In Pacific white shrimp, replacing 10–20% of fishmeal protein with BSFL meal reduced the relative abundance of opportunistic genera such as *Vibrio*, *Photobacterium*, and *Candidatus Bacilloplasma* and increased *Bacillus* and *Pseudoalteromonas*, changes that coincided with improved survival following *Vibrio* parahaemolyticus challenge. In contrast, increasing replacement to 30% negatively affected intestinal morphology and immune-related gene expression. Similarly, antimicrobial lipid fractions isolated from BSFL exhibited minimum inhibitory concentrations as low as 0.09 and 0.38 mg mL−1 against multidrug-resistant *Aeromonas hydrophila* and *A. salmonicida*, respectively. Together, these findings indicate that favorable microbial and physiological responses are often observed within specific inclusion ranges, whereas excessive incorporation may compromise intestinal function.

The studies summarized in **Table 2** support the functional potential of insect-derived feeds, but they also demonstrate that the relationship between dietary inclusion and gastrointestinal benefit is not linear or universal. Significant improvements in microbial diversity, mucosal characteristics, digestive efficiency, immune responses, or pathogen resistance have been documented, but these outcomes are frequently endpoint-specific and dependent on inclusion level, ingredient processing, and host species. Therefore, experimentally demonstrated responses should be distinguished from proposed mechanisms attributed to individual insect-derived compounds. Although chitin, antimicrobial peptides, medium-chain fatty acids, and other bioactive fractions are plausible contributors, most *in vivo* studies evaluate whole insect meals containing multiple simultaneously active constituents. Establishing causal relationships between individual compounds, microbial metabolites, and host physiological responses will therefore require more targeted dose-response studies and experimental designs combining ingredient fractionation with microbiome, metabolomic, and host-response analyses.

All these findings indicate that the immunological effects of insect-based diets should be interpreted as part of an integrated host response involving epithelial cells, resident microbiota, microbial metabolites, and bioactive dietary compounds. Further studies employing standardized challenge models and multi-omics approaches will be essential to clarify the signaling pathways through which insect-derived bioactive compounds regulate intestinal immunity across livestock and aquaculture species.

## 6 Knowledge gaps and future perspectives

Despite the growing body of evidence supporting the use of insect-derived ingredients as functional feed components in livestock and aquaculture systems, several critical knowledge gaps remain unresolved. Although current studies consistently suggest that insect-based diets can modulate gut microbiota composition and improve gastrointestinal physiology, important methodological, mechanistic, and translational limitations still prevent the establishment of standardized nutritional recommendations and the full integration of insect-derived ingredients into precision animal nutrition strategies (**Figure 4**).

**Figure 4:**
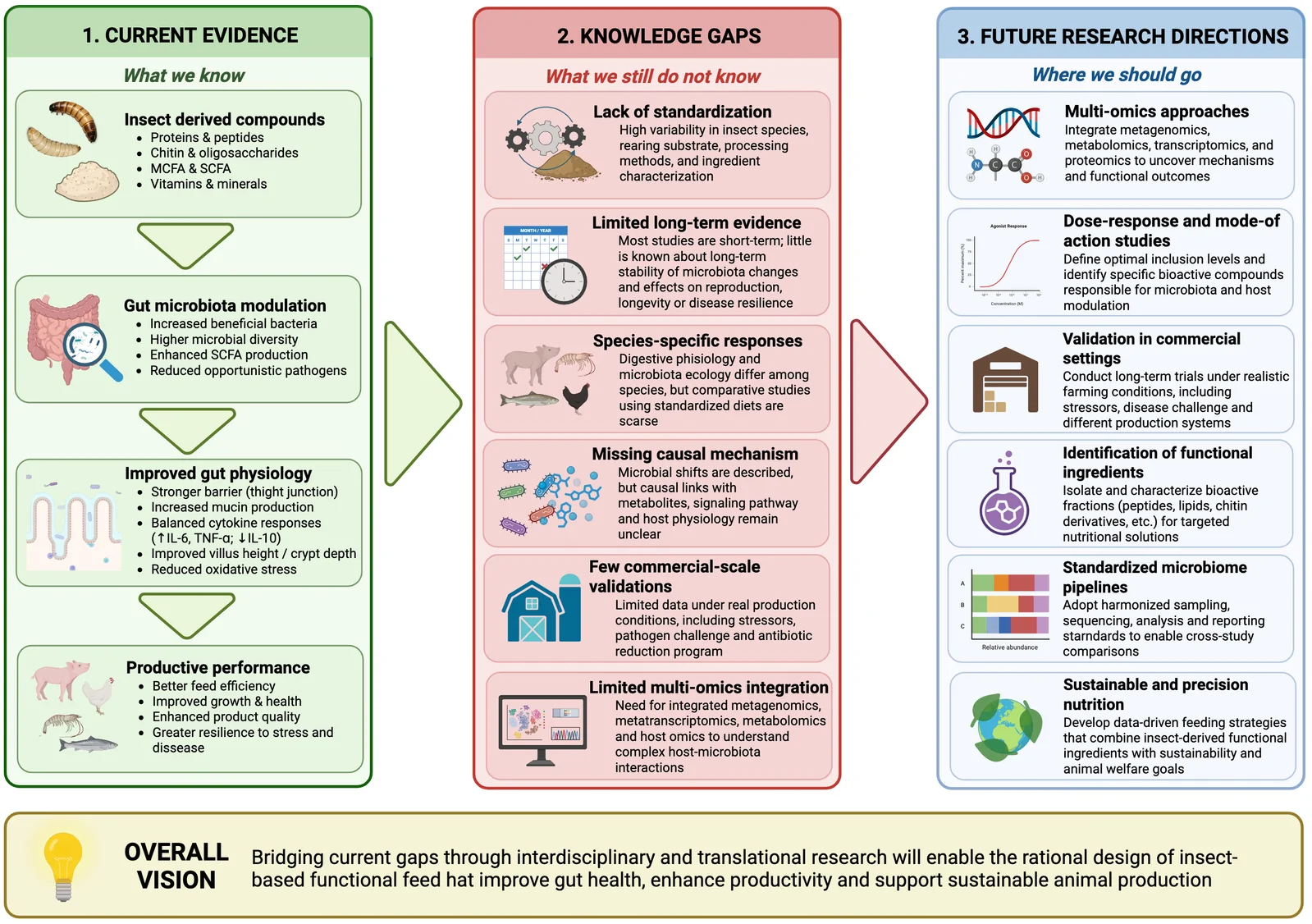
Conceptual framework summarizing current evidence, major knowledge gaps, and future research directions for insect-based diets and their impact on gut microbiota and host physiology in livestock and aquaculture.

One of the most important limitations in the current literature is the high degree of experimental heterogeneity among studies (Sogari et al., 2019). Variability in insect species, developmental stage, rearing substrate, processing technology, dietary inclusion level, host age, physiological status, and duration of feeding trials makes direct comparison between experiments difficult. For example, studies using BSFL meal often differ substantially in lipid content depending on whether the material is full-fat, partially defatted, or hydrolyzed, which may significantly influence both digestibility and microbiota responses (Barragán-Fonseca et al., 2017). Similarly, differences in thermal processing or drying methods may alter protein denaturation, chitin accessibility, peptide stability, and bioactive compound availability (Hawkey et al., 2021).

Future studies would benefit from adopting minimum reporting standards for insect-derived feed ingredients and experimental feeding protocols. Ingredient characterization should specify the insect species, developmental stage, rearing substrate, processing conditions, proximate composition, chitin content, and fatty acid profile, together with the dietary inclusion level and the conventional ingredient being replaced. Feeding trials should additionally report relevant characteristics of the animal model, including species, age or developmental stage, experimental duration, and dietary formulation. Where feasible, studies addressing gastrointestinal physiology should incorporate a comparable core set of outcomes, such as microbial composition, fermentation metabolites, intestinal morphology, mucin production, epithelial barrier-related markers, and inflammatory and oxidative indices. Greater consistency in reporting these variables would facilitate comparisons among studies and help distinguish responses attributable to insect species, ingredient composition, processing, or dietary inclusion level.

A second major gap concerns the limited mechanistic understanding of how specific insect-derived compounds influence microbial ecology and host physiology. Although chitin, AMPs, and MCFA have been proposed as key modulators of gut microbiota–host interactions, the precise biological pathways involved remain poorly characterized (Negash, 2022). Most available studies rely on taxonomic descriptions of microbiota shifts without identifying the functional metabolic consequences of these microbial changes. For example, increases in common bacterial genera are frequently reported, but their direct contribution to metabolite production, epithelial signaling, immune regulation, or nutrient metabolism is rarely explored (Biasato et al., 2018). Integrative approaches combining metagenomics, metatranscriptomics, metabolomics, and host transcriptomic analysis will be essential for identifying causal microbiota-mediated mechanisms.

Another important limitation is the relatively short duration of most feeding trials. Many published studies evaluate insect-based diets over periods ranging from a few weeks to a single production cycle, which may capture acute microbial adaptations but fail to reflect long-term physiological consequences (Su et al., 2025; Dabbou et al., 2021). Questions remain regarding the stability of microbiota changes over time, microbial resilience following dietary withdrawal, cumulative effects on intestinal development, and potential influences on reproductive performance, longevity, or disease susceptibility. Longitudinal studies covering multiple developmental stages would significantly improve our understanding of sustained host adaptation.

Species-specific variability also remains insufficiently characterized. Although poultry, swine, fish, and, to a lesser extent, crustaceans have shown promising responses to insect-based diets, comparative studies using standardized formulations across species are still scarce. Because digestive anatomy, microbial ecology, immune physiology, and nutrient metabolism differ substantially among host species, responses to insect-derived bioactive compounds may be highly context-dependent. Comparative physiology approaches may help identify conserved biological mechanisms while supporting the development of species-specific feeding strategies.

In addition, most available research has focused on healthy animals under controlled experimental conditions. Much less is known about how insect-based diets perform under commercial production stressors, including pathogen exposure, heat stress, transportation, social competition, or antibiotic reduction programs. Evaluating insect-derived ingredients under realistic production challenges could reveal their true functional value as gut health-promoting interventions (Sogari et al., 2019).

From an applied perspective, future research should move beyond evaluating insect meals solely as alternative protein sources and instead focus on identifying specific bioactive fractions with targeted physiological functions. Precision extraction of chitin derivatives, AMPs, lipid fractions, or fermented insect ingredients may open new opportunities for designing next-generation functional feeds capable of modulating gut microbiota, enhancing mucosal resilience, and improving sustainable animal production (Sogari et al., 2019; Su et al., 2025; Biasato et al., 2024; Alhasyani et al., 2025).

Ultimately, the integration of insect-derived functional ingredients into animal nutrition will depend on a deeper mechanistic understanding of microbiota–host interactions, robust species-specific validation, and the development of standardized industrial processing technologies capable of preserving bioactive functionality while ensuring economic scalability (Su et al., 2025; Biasato et al., 2024; Alhasyani et al., 2025).

## 7 Conclusions

The evidence reviewed throughout this manuscript demonstrates that their biological effects extend beyond nutrient supply and arise from the integrated action of several bioactive components—including chitin, antimicrobial peptides, medium-chain fatty acids, micronutrients, antioxidant compounds, and other functional metabolites—that collectively influence gut microbiota–host interactions. Across poultry, swine, fish, and crustaceans, insect-based diets have frequently been associated with favorable modifications of intestinal microbial communities, including increased bacterial diversity, enrichment of SCFA-producing microorganisms, and reductions in selected opportunistic pathogens. However, the direction and magnitude of these responses vary considerably according to the insect ingredient, processing conditions, dietary inclusion level, host species, and experimental setting. The evidence suggests that these physiological benefits arise from coordinated interactions among microbial metabolism, epithelial signaling pathways, and host immune regulation rather than from the action of any individual bioactive compound. Nevertheless, the current literature remains characterized by substantial heterogeneity in insect species, rearing conditions, processing technologies, dietary inclusion levels, animal models, and experimental methodologies. This variability limits direct comparisons among studies and highlights the need for standardized experimental protocols and mechanistic investigations capable of identifying causal biological pathways. Future research should prioritize multi-omics approaches, functional metabolomics, dose–response studies, and long-term validation under commercial production conditions to identify the specific insect-derived compounds responsible for gut health benefits and to establish evidence-based recommendations for their use. The successful integration of these approaches into precision animal nutrition will depend on continued interdisciplinary research linking insect biology, microbiology, gastrointestinal physiology, and feed technology.
